# LKB1 is a DNA damage response protein that regulates cellular sensitivity to PARP inhibitors

**DOI:** 10.18632/oncotarget.12334

**Published:** 2016-09-29

**Authors:** Yi-Shu Wang, Jianfeng Chen, Fengmei Cui, Huibo Wang, Shuai Wang, Wei Hang, Qinghua Zeng, Cheng-Shi Quan, Ying-Xian Zhai, Jian-Wei Wang, Xiang-Feng Shen, Yong-Ping Jian, Rui-Xun Zhao, Kaitlin D. Werle, Rutao Cui, Jiyong Liang, Yu-Lin Li, Zhi-Xiang Xu

**Affiliations:** ^1^ Key Laboratory of Pathobiology, Ministry of Education, Norman Bethune College of Medicine, Jilin University, Changchun, Jilin 130021, China; ^2^ Division of Hematology and Oncology, Comprehensive Cancer Center, University of Alabama at Birmingham, Birmingham, AL 35294, USA; ^3^ Department of Pharmacology and Experimental Therapeutics, Boston University, School of Medicine, Boston, MA 02118, USA; ^4^ Department of Systems Biology, UT MD Anderson Cancer Center, Houston, TX 77030, USA

**Keywords:** DNA damage, homologous recombination, LKB1, sensitization, PARP inhibitor

## Abstract

Liver kinase B1 (LKB1) functions as a tumor suppressor encoded by *STK11*, a gene that mutated in Peutz-Jeghers syndrome and in sporadic cancers. Previous studies showed that LKB1 participates in IR- and ROS-induced DNA damage response (DDR). However, the impact of *LKB1* mutations on targeted cancer therapy remains unknown. Herein, we demonstrated that LKB1 formed DNA damage-induced nuclear foci and co-localized with ataxia telangiectasia mutated kinase (ATM), γ-H2AX, and breast cancer susceptibility 1 (BRCA1). ATM mediated LKB1 phosphorylation at Thr 363 following the exposure of cells to ionizing radiation (IR). LKB1 interacted with BRCA1, a downstream effector in DDR that is recruited to sites of DNA damage and functions directly in homologous recombination (HR) DNA repair. LKB1 deficient cells exhibited delayed DNA repair due to insufficient HR. Notably, LKB1 deficiency sensitized cells to poly (ADP-ribose) polymerase (PARP) inhibitors. Thus, we have demonstrated a novel function of LKB1 in DNA damage response. Cancer cells lacking LKB1 are more susceptible to DNA damage-based therapy and, in particular, to drugs that further impair DNA repair, such as PARP inhibitors.

## INTRODUCTION

Genomic instability is a hallmark of cancer development and progression, and the most common form for genomic instability is the cellular accumulation of DNA damage. Such damage may arise from environmental insults such as ultraviolet radiation (UV), ionizing radiation (IR), exogenous chemicals and biological genotoxins, as well as endogenous sources, which lead to a variety of nucleotide modifications and DNA strand breaks [1, 2]. To combat these insults and maintain cellular homeostasis, cells have evolved a DNA repair network referred to as the DNA damage response (DDR), which includes complex signaling processes that sense, signal and repair DNA lesions [3]. As a result, cell cycle checkpoints and repair machinery proteins are activated [3]. It is well understood that mutations in DDR genes can result in a number of genomic instability syndromes that often lead to a heightened predisposition to cancers. For instance, germline mutations in breast cancer susceptibility gene 1/2 (*BRCA1/2*) and the Fanconi anemia (FA) genes are associated with increased susceptibility of breast cancer [2, 4–7]. In contrast, the molecular basis of genomic instability in sporadic cancers is not as well defined. Therefore, efforts to identify DNA repair genes, whose mutations lead to genomic instability in sporadic cancers, are still needed to further the knowledge of cancer development and progression as well as to develop more effective therapeutic regimens.

Liver kinase B1 (*LKB1*), also known as serine/threonine kinase 11 (*STK11*), was originally identified as a susceptibility gene of Peutz-Jeghers syndrome (PJS), an inherited disorder characterized by gastrointestinal tract polyps and predisposition to developing cancers [8–10]. Inactivating mutations in *LKB1* also lead to predisposition to sporadic cancers without PJS, such as lung adenocarcinomas, ovarian, breast, pancreatic, and cervical cancers [10–15]. Physiologically, LKB1 has a broad range of cellular functions involved in embryogenesis, cell polarity, energy metabolism, cell cycle, and apoptosis [16–25]. A few recent reports showed that LKB1 may also play a role in the maintenance of hematopoietic stem cells by balancing hematopoiesis and blood cell apoptosis [26–28]. When activated, LKB1 forms a heterotrimeric complex with two accessory subunits, the pseudokinase Ste20-related adaptor protein (STRAD) and the scaffolding protein MO25, and phosphorylates at least 13 members of the AMP-activated protein kinase (AMPK) superfamily [19, 29].

Analysis of the LKB1 protein sequence and structure has shown that LKB1 Thr 363/366 (Thr 363 in human, Thr 366 in mouse) lies in an optimal phosphorylation motif for ataxia telangiectasia mutated kinase (ATM), ATM- and rad3-related kinase (ATR), and DNA-dependent protein kinase (DNA-PK) [30]. Fernandes et al found that wild-type ATM displays a DNA damage–induced association with LKB1, BRCA1 and p53 [31]. Moreover, Sapkota et al reported that the phosphorylation of LKB1 at Thr 363/366 was triggered following the exposure of cells to IR, and that DNA damage-activated ATM kinase mediated this phosphorylation effect [32]. We and other groups recently found that LKB1 participates in ROS- and IR-induced DDR [33–35]. However, the precise mechanisms by which LKB1 promotes DNA repair and the significance of LKB1-induced DDR on cancer therapeutic are largely unknown.

In the present study, we investigated the role of LKB1 in IR-induced DDR and DNA repair, determined the impact of LKB1 deficiency on homologous recombination (HR) DNA repair, and validated a therapeutic strategy by targeting LKB1 deficient cancer cells. Our explorations suggest that depletion of LKB1 impairs DNA repair capability and sensitizes cells to DNA damaging agents in particular, to drugs that further impair DNA repair, such as PARP inhibitors.

## RESULTS

### LKB1 is involved in DDR

**Figure 1:**
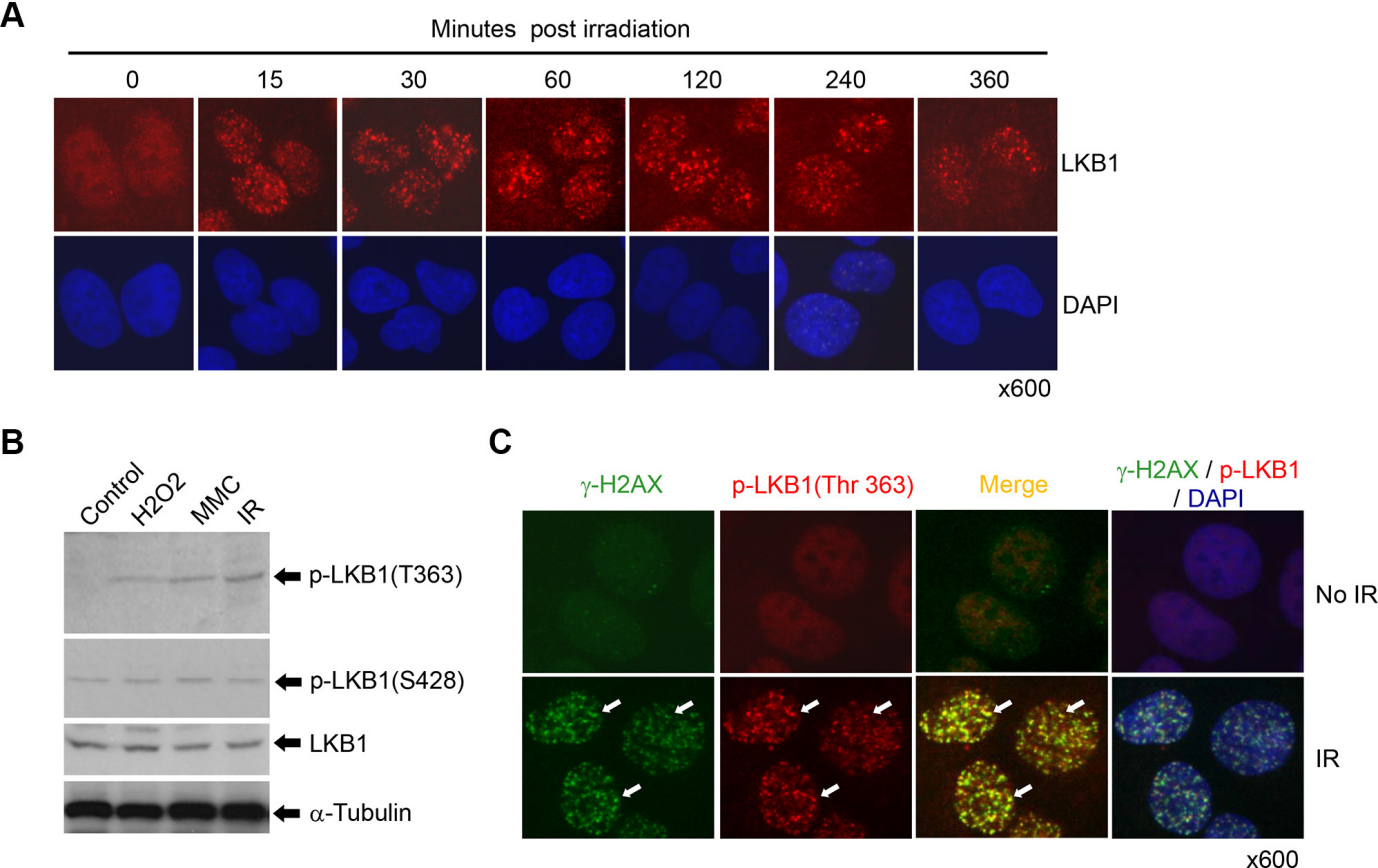
LKB1 is a DNA damage response protein (**A**) U2OS cells were treated with or without 5 Gy of γ-irradiation. The cells were fixed and stained with a monoclonal antibody against LKB1 at different time points after the irradiation. (**B**) U2OS cells were treated with 200 μM H2O2, 100 ng/ml mitomycin C (MMC), or 10 Gy γ-irradiation for 6 h. Western blot was performed to detect phosphorylated LKB1 (T363 and S428). a-Tubulin served as a loading control. (**C**) U2OS cells were exposed to 10 Gy γ-irradiation for 15 minutes. Cells were double-stained with monoclonal antibody to γ-H2AX and polyclonal antibody to phospho-LKB1 (T363). The white arrows show the co-localization of the two proteins.

DDR proteins typically form nuclear foci in response to DNA damage [4, 32]. To determine whether LKB1 plays a role in the DDR, we first examined the sub-cellular localization of LKB1 following the exposure of cells to IR. Nuclear LKB1 was detected in the majority of U2OS cells after IR, with the number and intensity of LKB1 foci increasing acutely following IR (Figure 1A). In contrast, there were very few nuclear LKB1 foci in cells not exposed to IR. Instead, the protein was distributed in the cytoplasm and nucleoplasm of untreated cells with a relative enrichment in nucleoplasm (Figure 1A). These observations indicate that LKB1 is involved in regulation of DDR.

Previous studies have reported that the phosphorylation of LKB1 at Thr 363 was triggered following the exposure of cells to IR [31]. Our current study validated that LKB1 was indeed phosphorylated at T363 after IR and other DNA damaging treatments (Figure 1B). To determine whether LKB1 phosphorylation (T363) responded to DNA damage to form IR-induced foci (IRIF), we irradiated U2OS cells and stained the cells with anti-phospho-LKB1 (T363) antibody. As shown in Figure 1C, phospho-LKB1 (T363) formed strong IRIF as early as 15 minutes after IR (Figure 1C). In contrast, phosphorylated LKB1 at S428, another phosphorylation site which is phosphorylated by PKA and p90RSK [36], was not altered by IR (data not shown and see results below), suggesting that phospho-LKB1 (T363) is a specific response to DNA damage. Moreover, phospho-LKB1 (T363) colocalized with phospho-histone H2A.X (γ-H2AX), a marker for DSB DNA damage (Figure 1C). These data further indicate that LKB1 is a DDR protein and that phospho-LKB1 (T363) may be one of the principal modification forms of LKB1 that respond to the DDR.

### LKB1 is phosphorylated by and co-localizes with ATM

**Figure 2:**
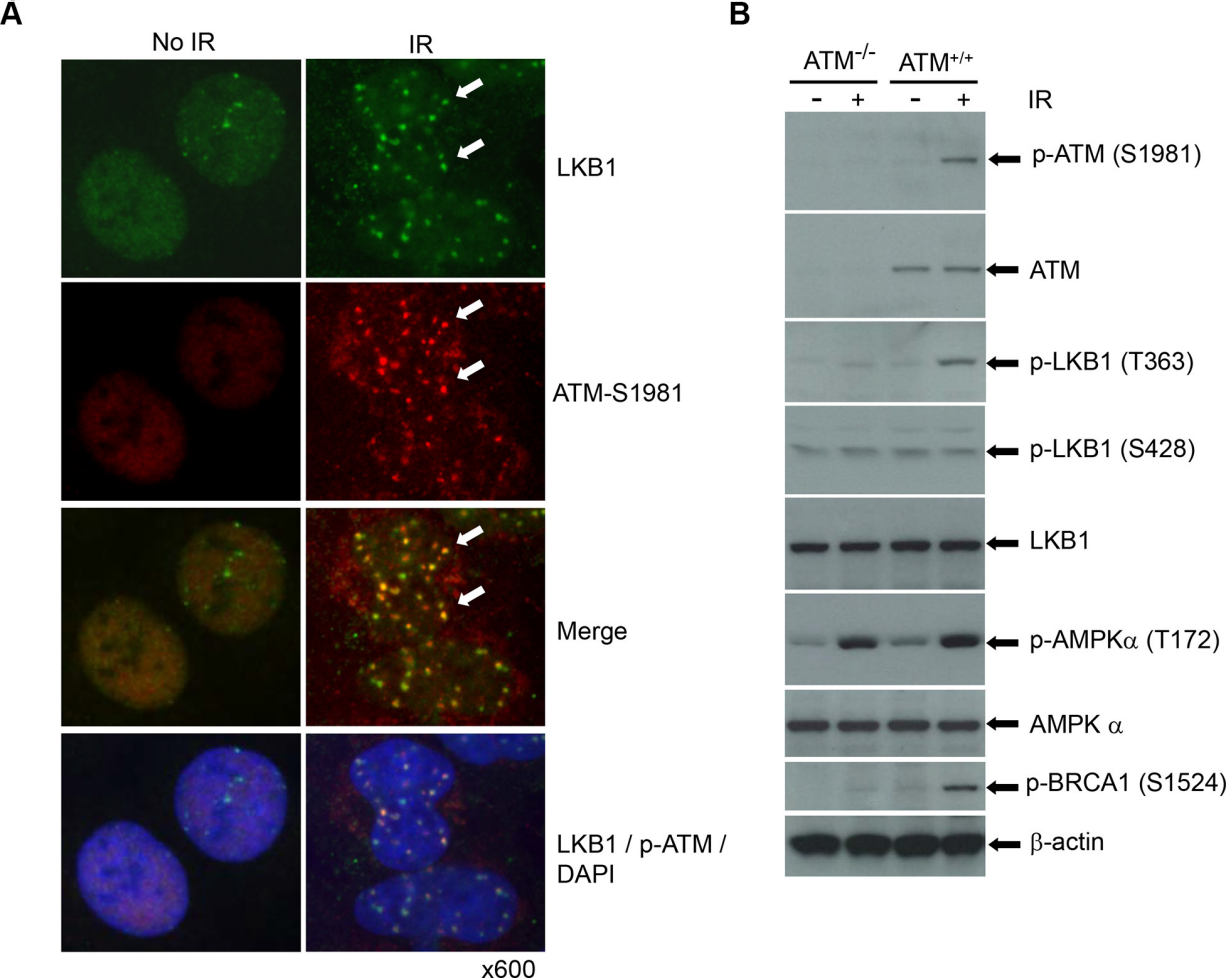
LKB1 is phosphorylated by and co-localizes with ATM (**A**) U2OS cells were treated with or without 5 Gy γ-irradiation. The cells were fixed and double-stained with LKB1 (green) and activated form of ATM (phospho-ATM S1981) (red) after 6 h post-irradiation. The white arrows show the co-localization of the two proteins. (**B**) Wild-type and ATM deficient cells were treated with or without 5 Gy IR. Thirty μg whole cell extracts (WCEs) were used for the western blot analysis. β-actin served as a loading control.

To effectively protect the genome, DNA structural alterations must be detected promptly. Several independent molecular complexes have been reported to sense and signal different types of DNA damage [3, 4]. ATM and ATR are recruited to broken DNAs, interact with substrates, and activate DDR cascades [4]. Computerized analysis has shown that LKB1 possesses an optimal phosphorylation motif (T363 of human, T366 of mouse) for ATM, ATR, and DNA-PK [31]. To determine whether LKB1 interacts with ATM *in vivo*, we analyzed the colocalization of LKB1 and activated ATM (phospho-ATM at S1981) in U2OS cells treated with IR. As shown in Figure 2A, both LKB1 and phospho-ATM were distributed diffusely in the cells. However, LKB1 localized to DNA damage-induced foci and co-localized with phospho-ATM (S1981) in cells after exposure to IR (Figure 2A), suggesting that DNA damage re-organizes LKB1 and ATM into a complex and may potentiate their interaction.

To gain an insight into the interaction between LKB1 and ATM and validate the ATM-dependent phosphorylation of LKB1, we irradiated wild-type and ATM deficient cells and analyzed LKB1 phosphorylation in the cells. Consistent with previous reports, IR triggered phosphorylation of BRCA1, which was dramatically reduced in ATM deficient cells (Figure 2B). In line with above study, we found that a substantial phosphorylation of LKB1 at Thr363 in the ATM wild type cells after IR (Figure 2B). In contrast, ATM-deficient cells showed only a weak phosphorylation of LKB1 at T363 with IR treatment (Figure 2B). Phosphorylation of LKB1 at S428 remained similar regardless of the ATM status and treatment with IR (Figure 2B). Interestingly, IR treatment also induced a marked increase of phospho-AMPK (T172), an immediate target of LKB1 [19]. However, unlike LKB1-T363, responses of AMPK (AMPK-T172) to IR were induced at a similar extent in both wild-type and null ATM cells, suggesting that the activation of AMPK was ATM-independent. Taken together, these observations further prove that LKB1 is a DDR protein that may interact with ATM and be involved in ATM-mediated DDR cascades.

### LKB1 deficiency leads to a delayed DNA-damage repair

**Figure 3:**
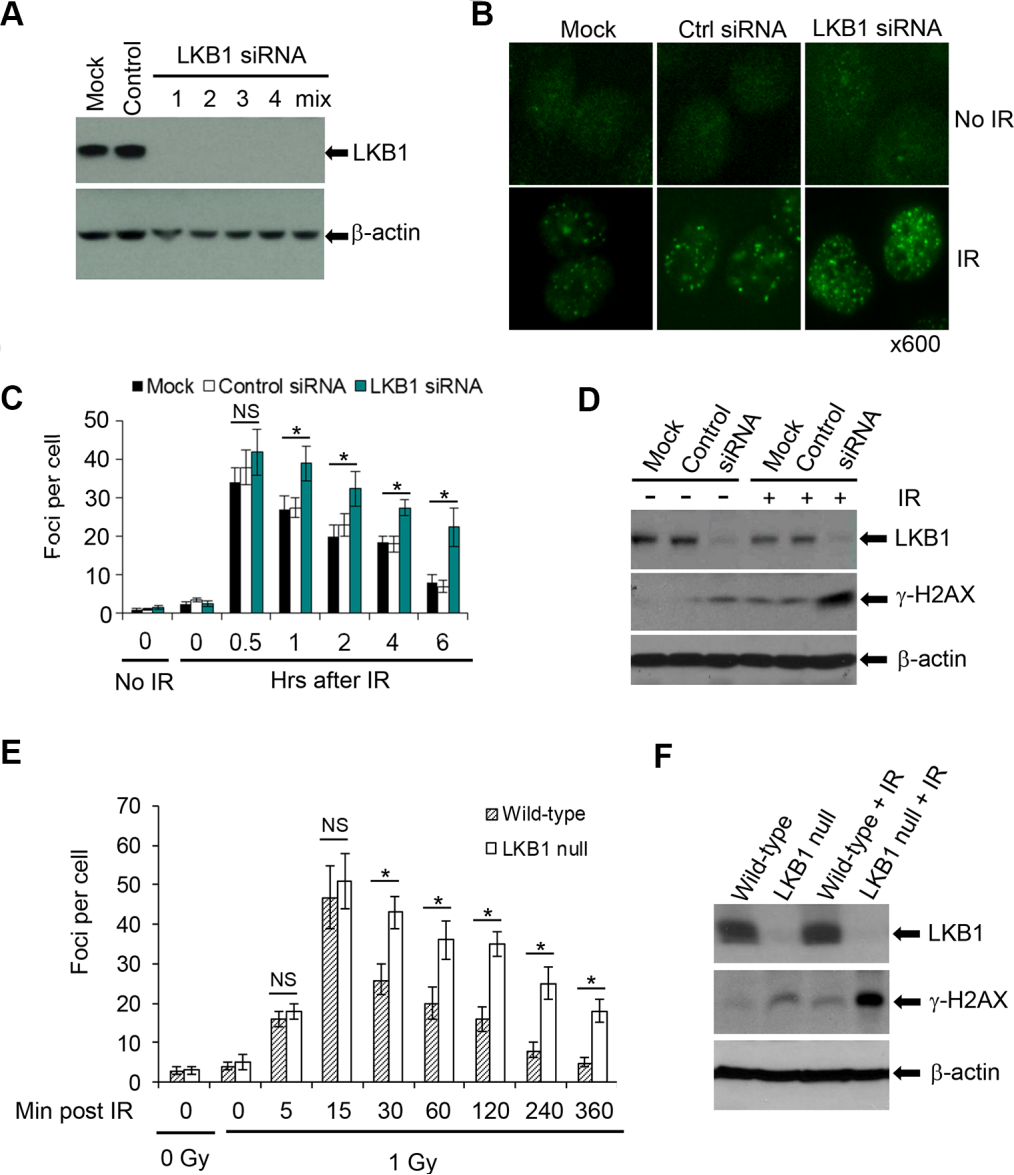
LKB1 deficiency delays DNA repair (**A**) Four siRNA duplexes and a smart pool were used to knock down endogenous LKB1 in U2OS cells. LKB1 level was detected by western blotting at 72 h after transfection. (**B**) γ-H2AX foci in mock, control, and LKB1 siRNA cells before or 6 h after 3 Gy irradiation shown by immunofluorescence assay. (**C**) The enumeration of γ-H2AX foci in LKB1 siRNA (smart pool) U2OS cells at different time points after 3 Gy of IR. For each time point, at least 100 cells were analyzed. Data shown are means ± SD from three independent experiments at each time point. *P < 0.01 as compared with mock and control groups. (**D**) Western blot detection of γ-H2AX in mock, control, and LKB1 siRNA cells before or 6 h after 3 Gy irradiation. Fifty μg protein was loaded. β-actin served as a loading control. (**E**) Wild-type and LKB1 null MEFs were treated with 1 Gy of γ-irradiation. Cells were fixed and stained with γ-H2AX at various time points post-irradiation. For each time point at least 100 cells were analyzed. Data shown are means ± SD from three independent experiments at each time point. *P < 0.01. NS, not significant. (**F**) Western blot detection of γ-H2AX in wild-type and LKB1 null MEFs before or 6 h after 1 Gy irradiation. Fifty μg proteins were loaded. β-actin served as a loading control. NS, not significant.

It is reported that γ-H2AX connotes the existence of DSBs, irrespective of their origin [37, 38]. γ-H2AX foci could serve as surrogates of DNA damage and γ-H2AX foci counts could be used to monitor the repair of IR-induced DSBs [37, 38]. To gain an insight into a possible role for LKB1 in the regulation of DSB repair, we knocked down endogenous LKB1 in U2OS cells with four duplexes of small interfering RNA (siRNA) and measured DSBs repair by counting γ-H2AX foci following IR. As shown in Figure 3A, every single duplex or pool of four duplexes of siRNA dramatically reduced the expression of LKB1. Although the number of γ-H2AX foci was similar in control siRNA and LKB1 siRNA cells within 30 min after IR, the number of γ-H2AX foci in control siRNA cells began to decrease within 60 minutes after IR while LKB1 siRNA cells had a constantly high number of γ-H2AX foci (Figure 3B and 3C). The average number of γ-H2AX foci per cell was almost two-fold higher in LKB1 siRNA-transfected cells than in control siRNA cells at 6 h after IR (Figure 3B and 3C). Immunoblot assay showed that both basal and IR-induced γ-H2AX levels were higher in LKB1 depleted cells than in control cells (Figure 3D). Collectively, these results suggest that LKB1 deficiency may delay DNA-damage repair and cause cells to maintain a higher level of γ-H2AX.

To further define the role of LKB1 in DNA-damage repair, we treated wild-type and LKB1 null MEFs with 1 Gy of IR and then examined the kinetics of γ-H2AX foci formation immediately after IR and at different time points thereafter. Both wild-type and LKB1 null MEFs showed about 16–18 γ-H2AX foci per cell 5 minutes after IR, which peaked at 47–51 γ-H2AX foci per cell 15 minutes after irradiation. The number of the γ-H2AX foci per wild-type cells gradually decreased to about 5 by 6 h after treatment (Figure 3E). In contrast, the γ-H2AX focus number remained higher in LKB1 null MEFs than in wild-type cells after 30 min post irradiation (Figure 3E). In addition, immunoblot detection also showed that LKB1 null MEFs possessed elevated levels of γ-H2AX both at basal and IR-induced levels when compared with wild-type cells (Figure 3F). Taken together, these data provide primary evidence for the involvement of LKB1 in DSB repair, and LKB1 deficiency leads to a delay of DSB repair.

### LKB1 deficiency reduces HR DNA repair efficiency

**Figure 4:**
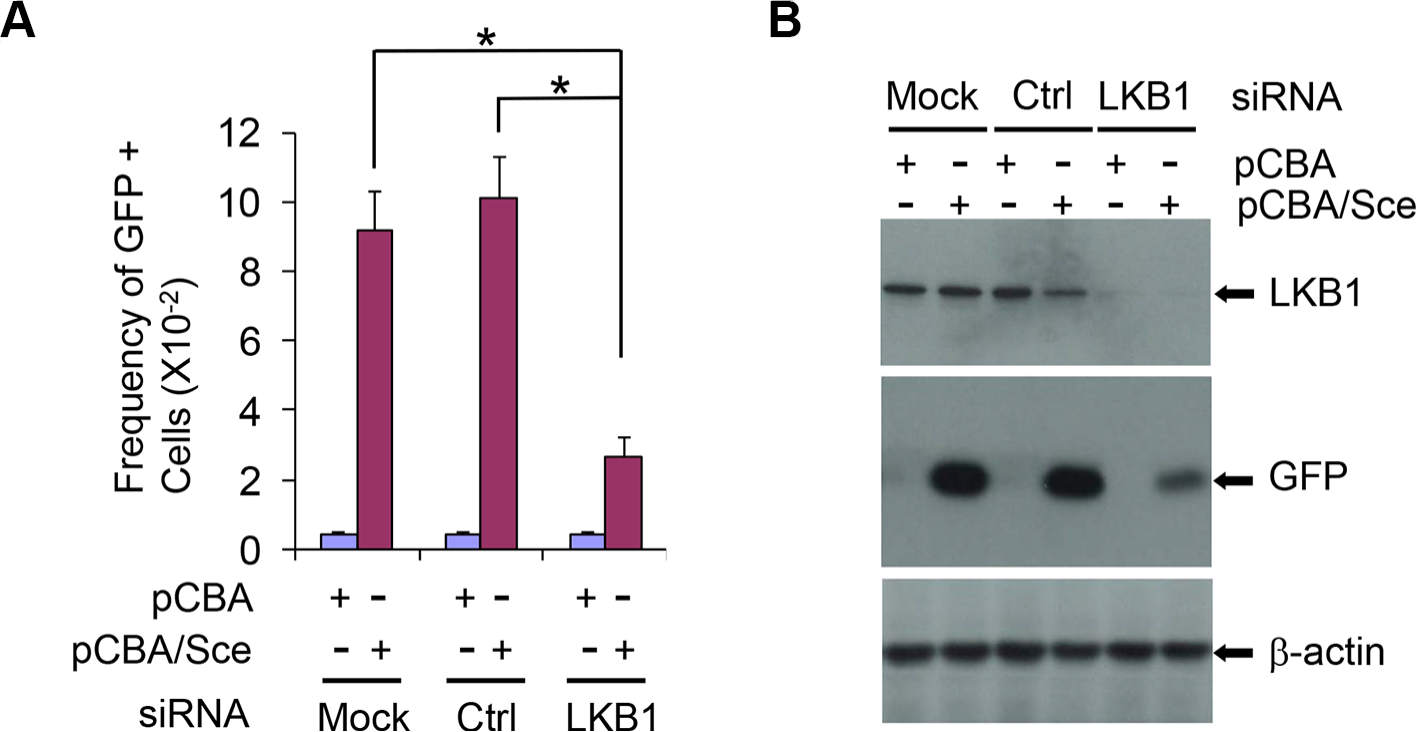
LKB1 affects HR DNA repair efficiency (**A**) The frequency of reconstitution of a GFP reporter gene within a chromosomally integrated plasmid substrate in control and LKB1 siRNA U2OS cells was examined. See details in *Materials and Methods* for determining the frequency of GFP-positive cells. Data shown are means ± SD from three independent experiments. *P < 0.01. (**B**) Western blotting analysis of GFP protein expressed in LKB1 siRNA, control siRNA, and mock cells after the homologous recombination. Fifty μg proteins were loaded. β-actin served as a loading control.

To define whether LKB1 is associated with HR DNA repair, we measured HR by examining the frequency of reconstitution of a GFP reporter gene within a chromosomally integrated plasmid substrate [39] in control and LKB1 siRNA cells. Knockdown of LKB1 (siRNA) led to a decreased amount of GFP positive cells (HR cells, 2–3% GFP positive cells), as compared with control or mock siRNA treatment (8–10% GFP positive cells, Figure 4A and Supplementary Figures S1, S2). Immunoblot assay showed that GFP protein expressed in LKB1 siRNA cells was also much lower than that in control or mock cells (Figure 4B). This effect was not due to the transfection efficiency in the absence of LKB1 since both the control and LKB1 siRNA cells exhibited a comparable GFP positive rate in control transfections (Supplementary Figure S3). Taken together, our data strongly suggest that LKB1 deficiency reduces HR DNA repair efficiency and delays the DNA damage repair.

### LKB1 interacts with BRCA1

**Figure 5:**
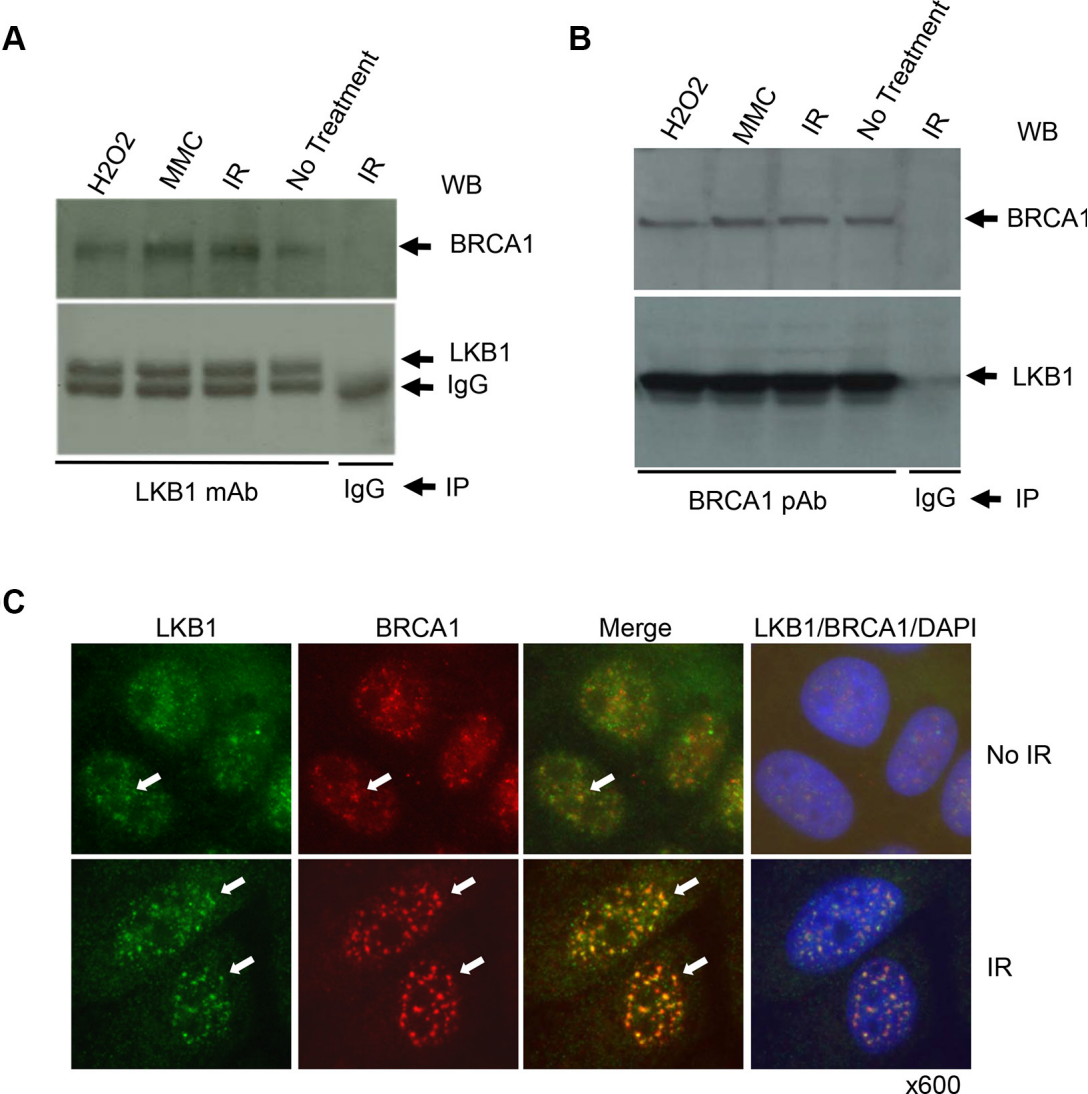
LKB1 is associated with BRCA1 (**A**), (**B**) U2OS cells were treated with 200 μM H2O2, 100 ng/ml MMC, or 10 Gy γ-irradiation, or left untreated. Whole cell extracts (WCEs) were isolated 6 h after the treatments. Five hundred μg WCE proteins were used for co-IP with anti-LKB1 monoclonal antibody (A) or anti-BRCA1 polyclonal antibody (B). The last lane is negative control for IP in which IgG was used instead of antibody. (**C**) U2OS cells were treated with or without 5 Gy γ-irradiation. The cells were harvested and immunostained with LKB1 (green) and BRCA1 (red) 6 h post irradiation. The white arrows show the co-localization of the two proteins.

BRCA1 is one of the most important DDR transducers and is essential for regulating DNA damage-induced cell cycle checkpoints and HR DNA repair [40]. It was reported that both LKB1 and BRCA1 interact with the Brg1 subunit of the SWI/SNF chromatin-remodeling complex [20, 41], which has a direct role in DNA-damage repair in addition to its role in the regulation of gene transcription. To determine whether LKB1 and BRCA1 interact directly, we performed co-IP experiments with anti-LKB1 or anti-BRCA1 antibody using lysates from U2OS cells treated with different DNA damaging agents, such as IR, H_2_O_2_, and mitomycin C (MMC). As shown in Figure 5A and 5B, BRCA1 and LKB1 co-immunoprecipitated with each other in the cells regardless of the presence or absence of DNA damages, suggesting that LKB1 and BRCA1 may interact within the same complex.

Because BRCA1 localizes to sites of DNA breaks in cells exposed to DNA damaging treatments, we next checked the co-localization of LKB1 with BRCA1 before and after 5 Gy IR treatment. Both LKB1 and BRCA1 were mostly distributed within the nucleoplasm of cells before the treatment, and there were some instances of co-localization between the two proteins (Figure 5C). In stark contrast, IR enhanced IR-induced foci (IRIF) formation for both LKB1 and BRCA1 and induced the co-localization of the two proteins (Figure 5C). Collectively, these results indicate that LKB1 interacts with BRCA1 and may participate in HR DNA repair through the interaction with BRCA1.

### LKB1 deficiency sensitizes cells to DNA damaging treatments

**Figure 6:**
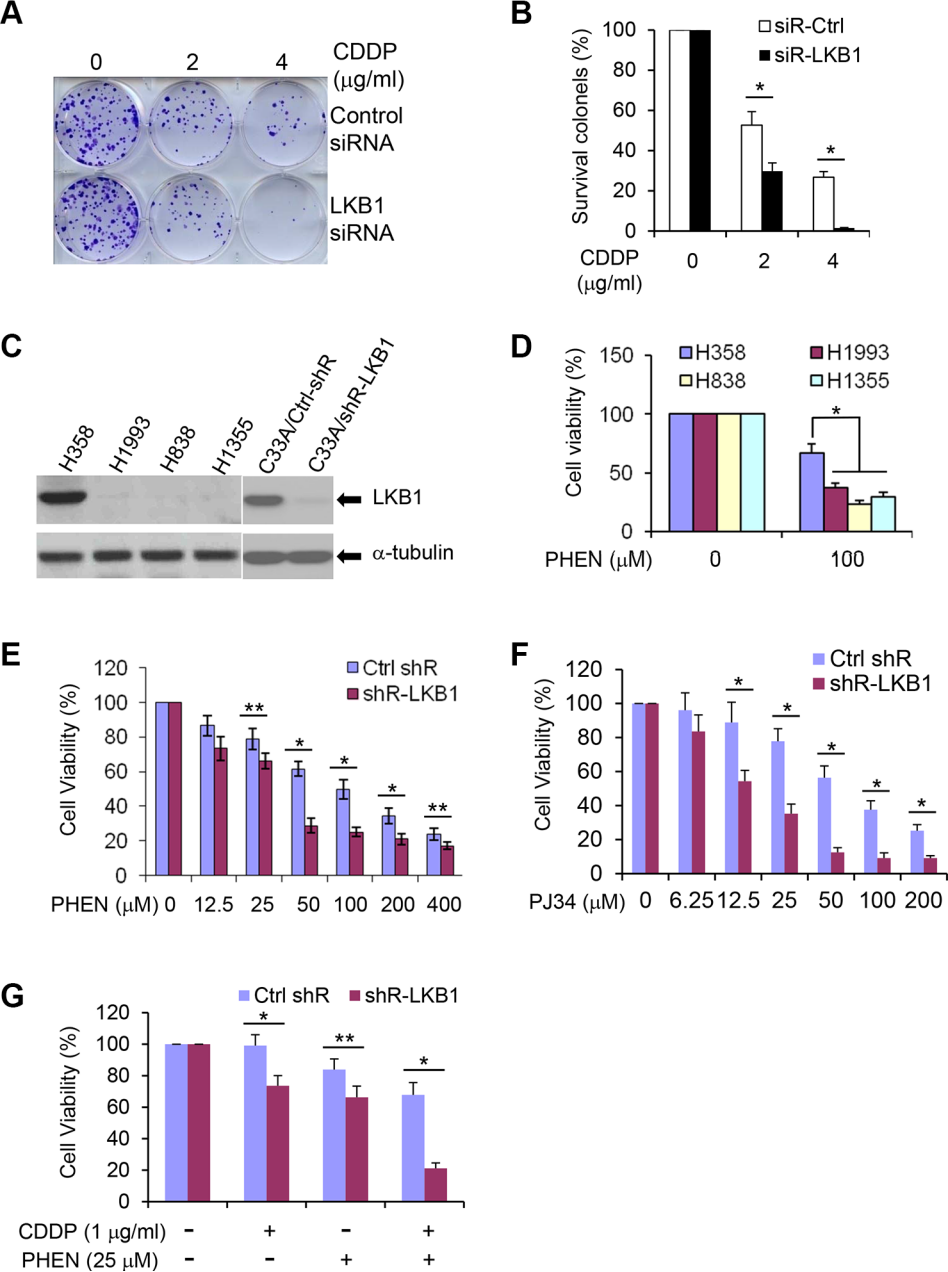
LKB1 deficiency sensitizes cells to DNA damage agents and PARP inhibitors (**A**) Colony formation assay in U2OS/Ctrl and U2OS/LKB1-siRNA cells exposed to CDDP for 1 h. Cells were allowed to grow for 14 days to form colonies and stained with 0.05% crystal violet. (**B**) Percentage of survival colonies in (A). Data are shown as means ± SD (*n* = 3). *P < 0.01. (**C**) Western blot detection of LKB1 in lung cancer cell lines, H358, H1993, H838, and H1355, and in cervical cancer cell line C33A with LKB1 shRNA knock-down.(**D**) H358, H1993, H838, and H1355 were exposed to 100 μM of PHEN, a PARP-1 inhibitor. Cell viability was determined by MTT assay 3 d after the treatment. Data are shown as means ± SD (*n* = 3). *P < 0.01. (**E**), (**F**) C33A/Ctrl-shR and C33A/shR-LKB1 cells were treated with different doses of PHEN (E) and PJ34 (F). Cell viability was determined by MTT assay 3 d after the treatment. Data are shown as means ± SD (*n* = 3). *P < 0.01; **P < 0.05. (**G**). C33A/Ctrl-shR and C33A/shR-LKB1 cells were treated with 1 μg/ml CDDP and 25 μM PHEN for 3 d. Cell viability was determined by MTT assay after the treatment. Data are shown as means ± SD (*n* = 3). *P < 0.01; **P < 0.05.

One of the consequences of defects in DNA repair is increased sensitivity to DNA damaging reagents. To determine whether LKB1 deficiency enhances cellular sensitivity to cytotoxic agents, we exposed LKB1 siRNA knock-down cells to cisplatin (CDDP) and examined the cell proliferation. In colony-forming assay, LKB1 siRNA-treated cells were more sensitive to CDDP treatment than were control siRNA cells (Figure 6A and 6B). With 4 ug/ml CDDP treatment, there was almost no colony formation in LKB1 deficient cells, whereas LKB1-intact cells had about 30% colony survival (Figure 6B). From these results, we conclude that LKB1 depletion compromises the ability of cells to respond to DNA damage and increases cell sensitivity to DNA damaging agent.

### LKB1 deficiency leads to cell susceptibility to PARP inhibitors

PARP-1 is the most abundant and active enzyme in the PARP family. It binds to both SSBs and DSBs, and its role in SSBs repair via the BER pathway has been most clearly elucidated [42]. Inhibition or down-regulation of PARP leads to the accumulation of SSBs, which are subsequently converted into DSBs at replication forks. Thus, cells deficient in HR DNA repair are extremely sensitive to PARP inhibitors [43–46]. The strong connection between LKB1 and HR DNA repair indicates that a lack of LKB1 may not only sensitize cells to standard platinum-based therapy as shown in Figure 6A, but also make the cells potentially susceptible to drugs that further hinder DNA repair, such as PARP inhibitors. To verify our hypothesis, we treated an LKB1 wild-type lung cancer cell line H358, and three LKB1 deficient lung cancer cell lines, H838, H1355, and H1993 (Figure 6C), with 6(5H)-phenanthridinone (PHEN), a PARP-1 inhibitor. As shown in Figure 6D, PHEN treatment markedly reduced cell viability of H838, H1355, and H1993 cells. However, LKB1 intact H358 cells were less sensitive to the treatment, suggesting that LKB1 deficiency may sensitize cells to PARP inhibitors.

To further validate the potential effect of LKB1 deficiency with PARP inhibitors, we depleted LKB1 expression in C33A cells, a cervical cancer cell line, using a lentivirus-mediated shRNA interference. The knockdown system efficiently reduced the expression of LKB1 in the cells (Figure 6C). LKB1 depletion was sufficient to sensitize the cells to PHEN and N-(6-Oxo-5,6-dihydrophenanthridin-2-yl)-(N, N-dimethylamino) acetamide hydrochloride (PJ34), another PARP-1 inhibitor that is in clinical trial (Figure 6E and 6F). In addition, combined application of CDDP and PHEN in LKB1 knock-down cells further deteriorated cell viability (Figure 6G). Taken together, these results strongly suggest that cancer cells lacking LKB1 are more susceptible to standard platinum-based therapy and to drugs that further impair DNA repair, such as PARP inhibitors.

## DISCUSSION

LKB1 functions as a tumor suppressor that physically associates with STRAD and MO25 to form a heterotrimeric complex [8–10, 47]. Upon STRAD binding, LKB1 translocates from the nucleus to the cytosolic compartment, where it plays an important role in controlling cell polarity, energy balance, protein synthesis, and cell cycle arrest [10, 47]. In contrast, the function of LKB1 in the nucleus is less well understood. A recent study showed that decreased nuclear LKB1 levels correlate with the metastasis of head and neck squamous cell carcinoma (HNSCC), suggesting a role of nuclear LKB1 in repressing HNSCC metastasis [48]. Our current study added a new function of nuclear LKB1 in HR DNA damage repair.

LKB1 has been demonstrated to be a target of ATM-mediated phosphorylation in response to DNA damage [31, 32]. In addition LKB1 appears to be required for the maintenance of chromosome stability and the LKB1 substrate, AMPKα2, was found to be recruited to DSBs in an LKB1-dependent manner to facilitate non-homologous end joining DNA damage [34]. Further LKB1 may also contribute to genomic stability in a manner upstream of BRCA1 [35]. In the current study, we showed that LKB1 formed DNA damage-induced nuclear foci and co-localized with DNA damage response proteins, such as ATM, γ-H2AX, and BRCA1, and that LKB1 directly interacted with BRCA1. Phosphorylation of LKB1 at Thr 363 occurred as early as 15 min after IR. In addition, we demonstrated that DNA repair was substantially delayed in LKB1-deficient cells due to the low efficiency of HR DNA repair. Thus, our study provides direct evidence for the characterization of LKB1 in DDR. Our data are consistent with the hypothesis that LKB1 deficiency may lead to an accumulation of DNA damage and an increase in genomic instability and tumorigenesis.

Intriguingly, oxidative damage was found to rapidly activate cytoplasmic ATM, where it appears to mediate LKB1 phosphorylation, leading to activation of the AMPK-TSC2-mTORC1 pathway [49]. However, the DNA-damage repair function of LKB1 against IR and chemotherapeutic agents took place mainly in the nucleus (Figures 1, 2), suggesting that the role of LKB1 in DNA damage repair is independent of its energy sensing function and is also different from its regulation on ROS.

One of the consequences of defects in DNA repair is increased radiation sensitivity, and hence extensive apoptotic cell death following IR. Consistent with this knowledge, we have demonstrated that LKB1 deficiency increased cell sensitivity to CDDP, and that colony formation and cell survival decreased in comparison with the behavior of LKB1 wild-type cells (Figure 6). Notably, LKB1 compromised cells are sensitive to the treatment of CDDP and PARP inhibitors (Figure 6G). PARP family proteins (mainly PARP-1 and PARP-2) participate in the physiological response against DNA damage and repair of SSB-induced DNA damage [50]. Lack of PARP activity through genetic modification or pharmacological inhibitors increases SSB counts. Unrepaired SSBs are then converted into DSBs at fork replication [50]; as a result, cells are flooded with DSBs and succumb to apoptotic cell death [51–53]. In most cell lines with intact DSB DNA repair mechanisms, treatment with PARP inhibitors at doses that successfully inhibit PARP activity does not cause cell death, providing an exquisite approach to specifically targeting cancer cells, especially those harboring mutant BRCA1 and BRCA2 [43–45, 54]. The effect of PARP inhibitors was then extended to tumors with other genes implicated in similar DNA repair pathways to BRCAs [55–59]. Our current research suggests that LKB1 responds to DNA damage and participates in HR-mediated DNA repair. Thus, it explained why the LKB1 deficient cells, such as cervical cancer cells and NSCLC cells, are highly sensitive to PARP inhibitors. Our results are also consistent with a recent report showing that exposure of HeLa cells, an LKB1 deficient cell line, to PARP inhibitors triggers cell apoptosis [60]. Exogenous expression of PARP-1 renders cells resistant to benzamide-induced apoptosis [60], suggesting that LKB1 deficiency may indeed be an “Achilles heel” for treatment with PARP inhibitors. With regard to a high mutation rate of LKB1 in a variety of cancers, such as cervical cancer and NSCLC, our exploit is extremely meaningful. If the results warrant in experimental animals, we anticipate that the clinical assessment of PARP inhibitors should be extended beyond those with BRCA mutations to a larger group of cancer patients with LKB1 mutations.

## MATERIALS AND METHODS

### Cell culture and reagents

Human U2OS (osteosarcoma) and wild-type- and LKB1-null mouse embryonic fibroblasts (MEFs), or wild-type- and ATM-deficient cells were maintained in Dulbecco's Modified Eagle Media (DMEM) supplemented with 10% fetal bovine serum (FBS, Thermo Scientific, Waltham, MA, USA) at 37°C in 5% CO2. Non-small cell lung cancer (NSCLC) cell lines, H358, H838, H1355, and H1993, were maintained in RPMI 1640 medium with 10% FBS. C33A (cervical cancer) cells were maintained in MEM with 10% FBS, 1% non-essential amino acids, and 1% sodium pyruvate. CDDP, PHEN, and PJ34 were purchased from Sigma-Aldrich (St. Louis, MO, USA).

### Antibodies

Polyclonal antibodies against phospho-LKB1(S428), phospho-BRCA1(S1524), phospho-H2AX (S139), phospho-AMPKa (T172), and AMPKa were purchased from Cell Signaling (Boston, MA, USA). Monoclonal antibodies against phospho-ATM (S1981) and phospho-H2AX (S139) were obtained from Millipore (Charlottesville, VA, USA). Phospho-LKB1 (T363/366) was purchased from ImmuQuest (North Yorkshire, UK). GFP, LKB1, and BRCA1 antibodies were also purchased from Santa Cruz Biotechnology (Santa Cruz, CA, USA). ATM and β-actin antibodies were obtained from Bethyl Laboratories (Montgomery, TX, USA).

### siRNA and transfection

Four siRNA duplexes for LKB1 were purchased from Dharmacon Inc. (Lafayette, CO, USA). The siRNA sequences will be provided by request. siRNA duplexes were transfected into the cells using DharmaFECT 2 Transfection Reagent (Dharmacon) according to the manufacturer's instruction.

### LKB1 stable knockdown using lentiviral short hairpin RNA

Five premade lentiviral LKB1 short hairpin RNA (shRNA) constructs and a negative control construct were purchased from Open Biosystems (Huntsville, AL, USA). Lentiviral helper plasmids (pCMV-dR8.2 dvpr and pCMV-VSV-G) were obtained from Addgene (Cambridge, MA, USA). Lentivirus stocks were prepared following the manufacturer's protocol. To select for the U2OS and C33A cells that were stably expressing shRNA constructs, cells were plated at sub-confluent densities and infected with 1 mL of virus-containing medium including 10 μg/mL polybrene. Selection with 0.375 to 1 μg/mL of puromycin was started 48 h after infection. After 4 weeks of selection, monolayers of stably infected pooled clones were harvested for use and cryopreserved.

### Ionizing radiation

Cells were ionize-irradiated (3.5 Gy/min) with a Nasatron generator in the presence of 10% FBS and then immediately transferred to a humidified incubator at 37°C in 5% CO_2_. After incubation for the indicated times, cells were harvested for immunofluorescent staining or western blotting analysis as described previously [61].

### Immunofluorescent staining

Cells grown on coverslips were fixed with 4% paraformaldehyde at room temperature for 15 min and then permeabilized with PBS containing 0.25% Triton X-100 for 10 min. The cells were blocked with 1% bovine serum albumin for 20 min before incubation with primary antibodies at room temperature for 1 h. After washing with PBS, cells were incubated with the secondary antibodies Alexa fluor–conjugated goat anti-mouse IgG or goat anti-rabbit IgG at room temperature for 1 h. After a final wash with PBS, coverslips were mounted with anti-fading mounting medium containing 4,6-diamidino-2-phenylindole (DAPI). Some cells on coverslips were washed twice in PBS, incubated in cytoskeleton buffer (PIPES pH 6.8, 100 mM NaCl, 300 mM sucrose, 3 mM MgCl_2_, 1 mM EGTA, 0.5% Triton X-100) for 5 min on ice, and then incubated in stripping buffer (10 mM Tris-HCl pH 7.4, 10 mM NaCl, 3 mM MgCl_2_, 1% Tween 20, 0.25% sodium deoxycholate) for 3 min on ice. The cells were washed three times in ice-cold PBS and then were fixed and processed as described above. The images were captured with an Olympus IX51 fluorescence microscope (Center Valley, PA, USA).

### Western blot

Cells were lysed in RIPA buffer (50 mM Tris–HCl pH 7.5, 150 mM NaCl, 1% Nonidet P-40, 0.5% sodium deoxycholate, 0.1% sodium dodecyl sulfate). Equal amounts of protein were separated by 6–15% SDS–PAGE followed by electrotransfer onto a polyvinylidene difluoride membrane (Thermo Scientific, USA). The membranes were blocked for 1 h with 5% nonfat milk and then incubated at room temperature with primary antibodies. The membranes were developed using an enhanced chemiluminescence detection system (GE Healthcare, USA).

### Co-immunoprecipitation assay

The RIPA extracts were pre-cleared with protein G-sepharose bead (Millipore), then incubated at 4°C overnight with appropriate antibodies. After the addition of fresh protein G-sepharose bead, the reaction was incubated for 4 h at 4°C with rotation. After five washes with buffer (50 mM PIPES, pH 7.5; 100 mM NaCl; 0.25 mM EGTA; 0.25 mM EDTA; 0.25% Triton X-100; 0.125% NP-40; 2.5% glycerol; protease inhibitors) and one wash with ice-cold PBS, precipitated proteins were dissolved in an equal volume of 2× SDS loading buffer and were then analyzed by western blotting using the appropriate antibodies.

### Colony formation assay

Cells (1 × 10^3^) were treated with CDDP at various concentrations for 1 h. After being rinsed with fresh medium cells were allowed to grow for 10–14 days to form colonies, which were fixed with cold methanol and stained with 0.05% crystal violet. The colonies containing more than 50 cells were counted. The fraction of surviving cells was calculated as the ratio of the plating efficiencies of treated cells to untreated cells. The mean ± S.D. from three independent experiments was determined.

### Cell viability assay

Six thousand cells were seeded into each well of 96-well plates and allowed to adhere overnight. Cell viability was determined 72 h after the treatment using the MTT assay. Plates were read with a Synergy H1 microplate reader (BioTek Instruments, Inc., Winooski, VT) at a wavelength of 530/620 nm.

### Homologous recombination DNA repair assay

A synthetic HR repair substrate system was developed and kindly provided by Dr. Maria Jasin from Memorial Sloan-Kettering Cancer Center (New York, NY). Three constructs are used in the system. In the DR-GFP plasmid, there are two separate GFP expression sequences. In the first GFP sequence, I-Sce I enzyme cognate sequence was inserted to the full GFP sequence. The second GFP sequence is an incomplete GFP sequence. Therefore, both of them will not show the fluorescence in normal conditions. When the I-SCE I is transfected to the DR-GFP positive cells, expressed SCE I digests the I-SCE I sequence in the DR-GFP plasmid to generate a double strand break. Thus, the broken GFP sequence forms a complete GFP reading frame via the HR with the second GFP sequence in the construct. The HR efficiency can then be determined by the proportion of GFP cells present. We transfected the DR-GFP plasmid to U2OS cells, and positive clone integrated with a single copy of reporter was identified as previously described. The cells were then selected with hygomycin to make a stable cell line of U2OS/DR-GFP. To evaluate homologous recombination repair of DSBs, U2OS/DR-GFP cells were either transfected with pEGFP (containing full-length GFP cDNA) or transfected with pCBA-SceI plasmid (containing full-length I-SceI expression sequences). Transient expression of I-SceI endonuclease generates a double-strand break at the integrated GFP gene sequences and stimulates HR repair. GFP signal was assayed at 2 days post-transfection by immunofluorescence microscopy. The frequency of recombination events was calculated from the frequency of GFP signal in U2OS-DRGFP cells transfected with I-SceI by subtracting the frequency of GFP signal in U2OS-DRGFP cells without transfection and dividing by the frequency of GFP signal in U2OS-DRGFP cells transfected with pEGFP.

### Statistical analysis

The student *t-test* was used for the statistical analysis. Differences were considered to be significant if the *p value* was < 0. 05.

## SUPPLEMENTARY MATERIALS FIGURES


