# Supplementary material for: LKB1 is a DNA damage response protein that regulates cellular sensitivity to PARP inhibitors
Source: Oncotarget. 2016 Sep 29;7(45):73389–401. doi: 10.18632/oncotarget.12334 (PMC5341986; doi:10.18632/oncotarget.12334)
Supplement: Supplementary file 1 [file oncotarget-07-73389-s001.pdf]

LKB1 is a DNA damage response protein that regulates cellular sensitivity to PARP inhibitors

Supplementary Materials

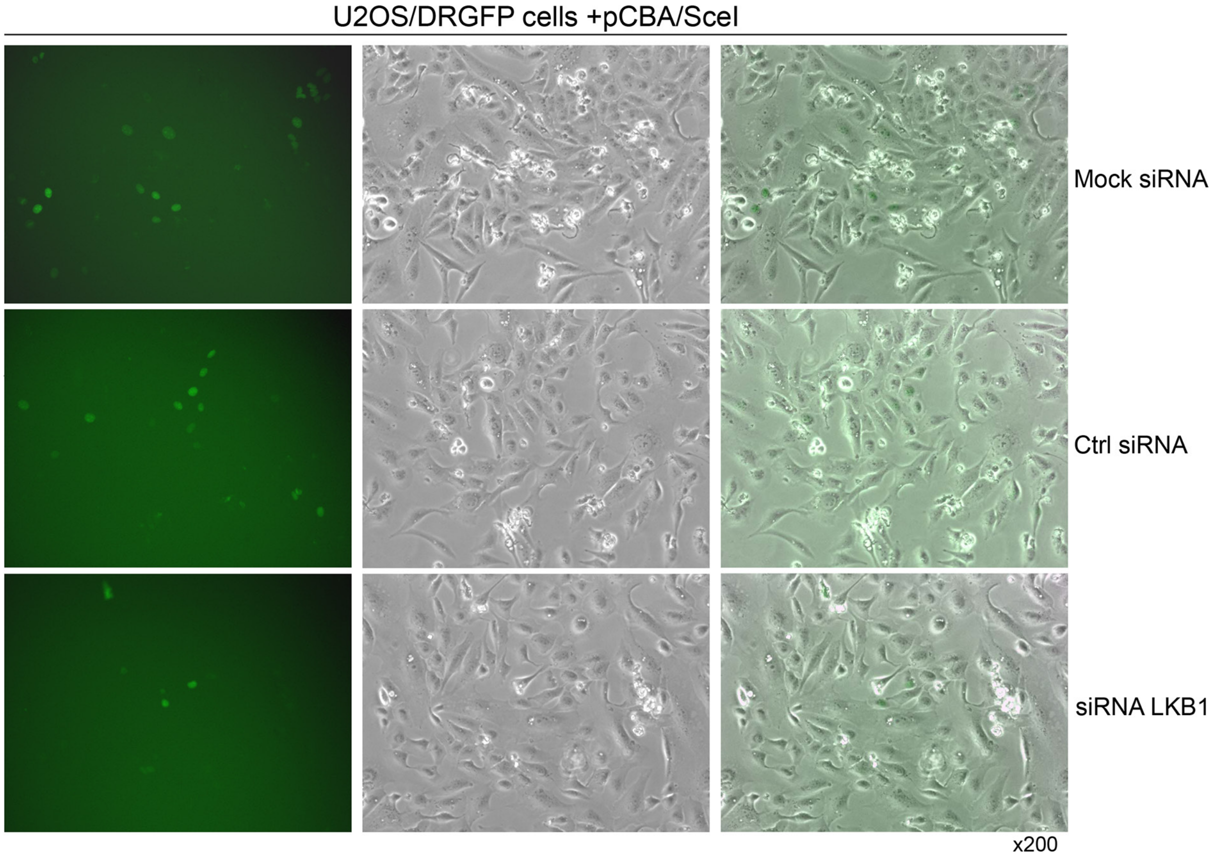

Supplementary Figure S1: LKB1 is associated with homologous recombination DNA repair. U2OS/DR-GFP cell line was established as described in *Materials and Methods*. The cells were transfected with pCBA/I-SCE I construct to make a single double strand break in the genome. The GFP fluorescence was assessed to determine the homologous recombination efficiency after the transfection. Representative fluorescence figures are shown. LKB1 siRNA cells exhibited a reduced GFP-positive cell number as compared with mock and control siRNA cells, suggesting that LKB1 deficiency compromises cell homologous recombination DNA repair.

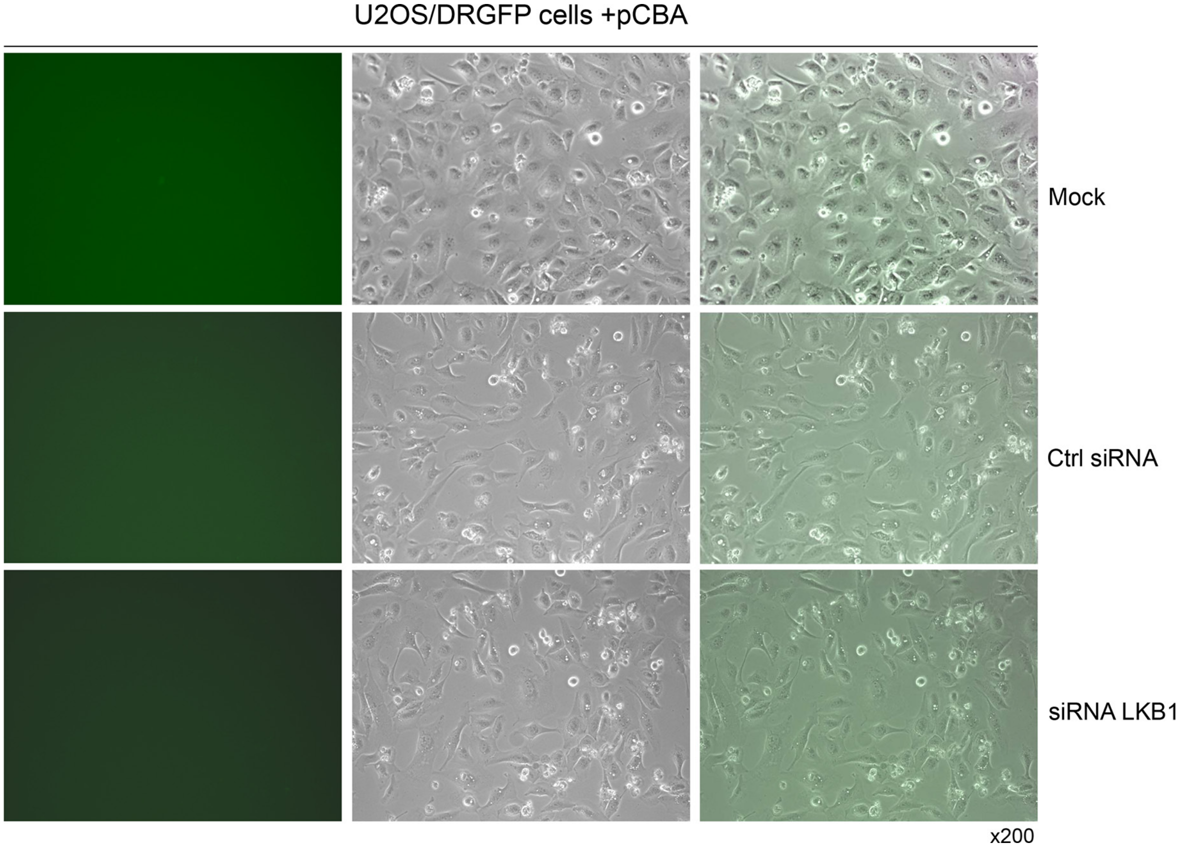

Supplementary Figure S2: LKB1-mediated homologous recombination DNA repair is DNA damage dependent. U2OS/DR-GFP cell line was established as described in *Materials and Methods* and in Supplementary Figure S1. Instead of the transfection of the cells with pCBA/I-SCE I construct to make a single double strand break in the genome as shown in Supplementary Figure S1, the cells were transfected with vector construct pCBA. The additional treatments were similar as in Supplementary Figure S1. Since there was no SCE I-mediated double strand break, SceGFP was unable to perform a homologous recombination with truncated iGFP to form a full length GFP. Thus, there was no fluorescence GFP signaling in all the groups.

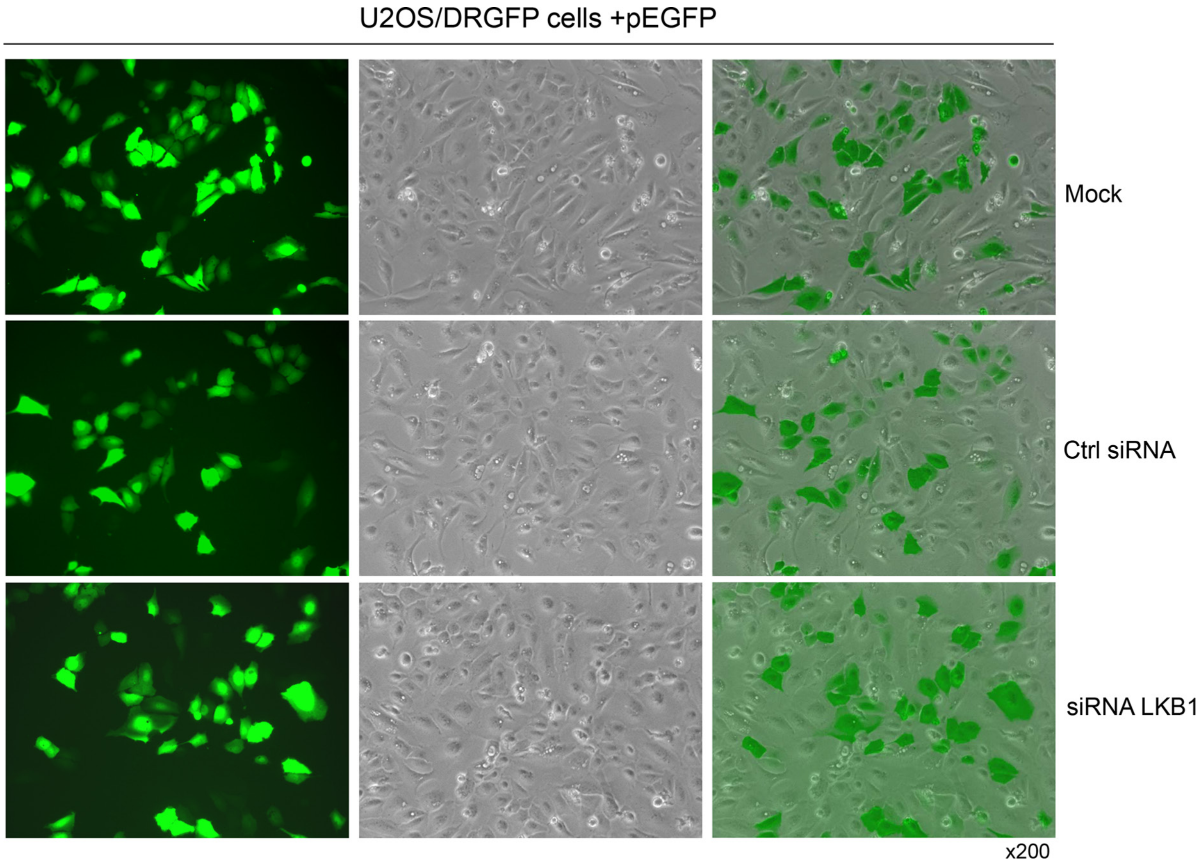

Supplementary Figure S3: LKB1 depletion-induced insufficiency in homologous recombination DNA repair is not due to the transfection efficiency. U2OS/DR-GFP cell line was established as described in *Materials and Methods* and in Supplementary Figures S1 and S2. The cells were transfected with pEGFP vector to validate the transfection efficiency in the cells. Representative fluorescence figures are shown. Cells treated with mock, control siRNA, and LKB1 siRNA exhibited a comparable GFP positive rate (63%, 56%, and 59%, respectively).
